# Multivariate versus traditional quantitative phase analysis of X-ray powder diffraction and fluorescence data of mixtures showing preferred orientation and microabsorption

**DOI:** 10.1107/S1600576722004708

**Published:** 2022-07-05

**Authors:** Mattia Lopresti, Beatrice Mangolini, Marco Milanesio, Rocco Caliandro, Luca Palin

**Affiliations:** a Università del Piemonte Orientale, Dipartimento di Scienze e Innovazione Tecnologica, Viale T. Michel 11, 15121 Alessandria, Italy; b Institute of Crystallography, CNR, via Amendola 122/o, 70126 Bari, Italy; c Nova Res s.r.l., Via D. Bello 3, 28100 Novara, Italy; Ecole National Supérieure des Mines, Saint-Etienne, France

**Keywords:** X-ray powder diffraction, quantitative phase analysis, Rietveld refinement, multivariate analysis, principal component analysis, X-ray fluorescence, preferred orientation, microabsorption

## Abstract

In materials and earth science, but also in chemistry, pharmaceutics and engineering, the quantification of elements and crystal phases in solid samples is often essential for a full characterization of materials. The most frequently used techniques for this purpose are X-ray fluorescence (XRF) for elemental analysis and X-ray powder diffraction (XRPD) for phase analysis. In both methods, relations between signal and quantity do exist but they are expressed in terms of complex equations including many parameters related to both sample and instruments, and the dependence on the active element or phase amounts to be determined is convoluted among those parameters. Often real-life samples hold relations not suitable for a direct quantification and, therefore, estimations based only on the values of the relative intensities are affected by large errors. Preferred orientation (PO) and microabsorption (MA) in XRPD cannot usually be avoided, and traditional corrections in Rietveld refinement, such as the Brindley MA correction, are not able, in general, to restore the correct phase quantification. In this work, a multivariate approach, where principal component analysis is exploited alone or combined with regression methods, is used on XRPD profiles collected on *ad hoc* designed mixtures to face and overcome the typical problems of traditional approaches. Moreover, the partial or no known crystal structure (PONKCS) method was tested on XRPD data, as an example of a hybrid approach between Rietveld and multivariate approaches, to correct for the MA effect. Particular attention is given to the comparison and selection of both method and pre-process, the two key steps for good performance when applying multivariate methods to obtain reliable quantitative estimations from XRPD data, especially when MA and PO are present. A similar approach was tested on XRF data to deal with matrix effects and compared with the more classical fundamental-parameter approach. Finally, useful indications to overcome the difficulties of the general user in managing the parameters for a successful application of multivariate approaches for XRPD and XRF data analysis are given.

## Abstract 2

A case study on the use of both multivariate statistics and traditional methods for ill-conditioned powder samples (microabsorption and preferred orientation) using X-ray fluorescence and diffraction data is presented. Indications and recipes on how to exploit multivariate methods to limit and overcome the difficulties of real-world industrial samples and control the analytical error are given.

## 1. Introduction

The quantification of elements and phases in solid-state materials represents a very important issue in many fields of science in both the academic and industrial world. X-ray powder diffraction (XRPD) and X-ray fluorescence (XRF) are widely used in this field to analyse crystalline phases and atomic elements, respectively. The advantages of X-ray-based techniques are many, since these techniques can be partially or totally nondestructive and probe relatively large amounts (grams) of samples with a statistical relevance. An additional advantage is measuring the sample ‘as received’, not altering its natural conditions. X-ray techniques can also be exploited in the field of portable instruments (Sarrazin *et al.*, 1998 ▸) for experiments under *in situ* (Eveno *et al.*, 2011 ▸) or *operando* (Urakawa, 2016 ▸) conditions. This approach was extended to its extreme, carrying out XRPD in the extraterrestrial world on the Moon (Vaniman *et al.*, 1992 ▸) and on Martian soil (Delhez *et al.*, 2003 ▸; Bish *et al.*, 2013 ▸).

Generally a whole profile fit approach is exploited, and a profile calculated from the atomic crystal structure of each pure component of the mixture is used to fit the whole experimental XRPD pattern in order to calculate the corresponding weight fractions, thus performing a Rietveld refinement (RR) (Rietveld, 1969 ▸). In XRF analysis (X-ray emission spectra), elements can be quantified by calibration with standards or by the generic fundamental parameter (FP) approach (Schönenberger *et al.*, 2012 ▸). Some software, such as *MAUD* or *TOPAS* (Lutterotti & Bortolotti, 2003 ▸; Coelho, 2018 ▸), can couple information from different techniques (*e.g.* XRPD, XRF, reflectivity experiments…) to exploit RR. Multivariate statistical analysis (MSA), requiring no or little *a priori* information, is a recently explored alternative (Caliandro *et al.*, 2013 ▸; Zappi *et al.*, 2019 ▸; Guccione *et al.*, 2021 ▸) to the above-cited traditional approaches (widely described in Appendix *A* [App appa]) of XRPD and XRF data analysis. The MultiFit regression procedure (Caliandro & Belviso, 2014 ▸) requires only pure phase profiles, while principal component analysis (PCA) is a completely blind approach (Jolliffe & Cadima, 2016 ▸); both approaches, available within *RootProf* (Caliandro & Belviso, 2014 ▸), a software (free for academic use) built specifically to manage XY profiles such as XRPD, XRF or other typical instrumental data, are the topic of the present contribution.

### 1.1. The potentialities and limitations of XRPD and XRF

The potentialities of XRPD in analytical chemistry were already envisaged in the early days of X-ray diffraction (Hull, 1919 ▸) and in its full development in the second part of the 20th century (Copeland & Bragg, 1958 ▸). More recently, new applications have emerged in specific utilizations, such as in the cultural heritage (Artioli *et al.*, 2003 ▸, 2017 ▸; Dooryhee & Colomban, 2008 ▸; Brunetti *et al.*, 2016 ▸) and pharmaceutical (Fawcett *et al.*, 2019 ▸) fields. This approach has been widely explored in recent decades (Madsen *et al.*, 2001 ▸; Scarlett *et al.*, 2002 ▸; De la Torre & Aranda, 2003 ▸; León-Reina *et al.*, 2009 ▸; Ufer & Raven, 2017 ▸; Raven & Self, 2017 ▸). The need for complete knowledge of the crystal structure was also overcome by the PONKCS (Scarlett & Madsen, 2006 ▸; Madsen *et al.*, 2019 ▸) approach, which is able to apply RR to partial or no known crystal structures.

One of the major critical issues in XRPD applications, especially phase quantification, is the tendency of microcrystals to be oriented along a preferred direction, which is favoured in the case of needle or platelet-like morphology (Dickson, 1969 ▸; Sitepu *et al.*, 2005 ▸; Monaco & Artioli, 2011 ▸). Preferred orientation (PO) causes biased intensities for the oriented phase (Madsen *et al.*, 2019 ▸). Moreover, in the presence of phases with large differences in linear absorption coefficient (LAC) and particle diameter in coarse powders, the heavily absorbing ones are underestimated as much as their particles are large, because of the effect known as microabsorption (MA) (Madsen *et al.*, 2019 ▸). This issue dramatically affects quantification, especially in the presence of particles of diameter above a few micrometres. MA in XRPD can be considered a matrix effect (ME), since it can severely affect the reliability of the results, in strict relation with sample composition and morphology. In fact, when MA is present, the relation between intensity of the signals and weight fractions can be lost, since it depends on the volume subjected to the incident flux in relation to particle size. In other words, the more heavily absorbing phases will be less penetrated by the X-rays, while phases with lower absorption coefficient are much more transparent and are more likely to exhibit ‘volume diffraction’ where the entire grain contributes to the diffraction process.

When constraining weight fractions to 1 according to equation (7) in Appendix *A* [App appa], MA causes an underestimation of the more absorbing crystalline phase and consequently an overestimation of the less absorbing phase. To mitigate this effect, costly and time-consuming procedures are required to prepare the sample for the measurement and mitigate MA. For instance, gentle milling allows the crystallites to be ground without inducing defect formation and crystallinity reduction. A widely used solution is the McCrone mill to reduce the particle size of the powder from a maximum of 0.5 mm particles to some micrometres, depending on the sample and grinding conditions required for quantitative and qualitative analytical methods, avoiding stress/strain/amorphization in the crystallites. Sieving can be useful, but the combination of milling and sieving can severely alter the sample, which is totally destructive, time consuming and not applicable in many fields. When MA and PO concur to affect diffracted intensities in solid mixtures, quantitative phase analysis (QPA) becomes even more complicated, if not impossible. Similarly, the main obstacle hindering quantification by XRF is referred to as the matrix effect (Bowers, 2019 ▸), again a signal enhancement and/or reduction induced by the presence of other elements in the analysed mixture. It is exacerbated if the sample is measured ‘as received’, without pearl fusion (time consuming and destructive).

Widespread solutions are the FP approach and algorithms based on the influence coefficients (Criss & Birks, 1968 ▸; Rousseau, 1984*a* ▸,*b* ▸; Willis & Lachance, 2004 ▸), which take into account the ME in XRF data, exploiting theoretical or empirical influence coefficients that are specific to each analyte–interferent pair. The full empirical calibration with known standard is an alternative but is limited in small concentration regions, with the additional limit of being sample specific and very time consuming. The problem becomes more complex when the sample is made of mixed inorganic and organic materials. The *K*α line of carbon can be measured both with high-end energy-dispersive XRF and with wavelength-dispersive XRF, but only if carbon is present in relatively high concentrations (*e.g.* above 50%) (Parus *et al.*, 2000 ▸). Moreover, below such a threshold, the measured intensity of the emission line of the heavy atom becomes independent of its weight fraction, making the analysis impossible (Grieken & Markowicz, 2001 ▸) unless the sample is diluted with a lighter element. The aim of this article is to find quick and reliable methods to process efficiently a large number of samples while limiting, as much as possible, sample preparation.

### 1.2. Multivariate statistical analysis

MSA is a collection of methods extensively used in analytical chemistry and, in particular, in the ‘-omics’ sciences (Sharaf *et al.*, 1986 ▸; Varmuza & Filzmoser, 2016 ▸), such as metabolomics and proteomics. In the MSA approach, the data (of any kind) are organized in matrices and analysed using algorithms that allow searching for correlations between the variables (Anderson, 2003 ▸). This approach ensures that important effects due to, for instance, the synergistic or antagonistic interactions (*i.e.* positive or negative correlations) between variables (for instance, intensities at different 2θ angles in the XRPD case), are efficiently and correctly identified. This multi-purpose approach is commonly exploited for classification, regressions and pattern recognition, in which unknown experimental domains are explored (Anderson, 2003 ▸; Johnson & Wichern, 2007 ▸). The peculiarity of MSA is the capability to extract efficiently the useful information, with background suppression and bias identification, possibly without or with very little *a priori* information.

PCA is a well known method for experimental error suppression applied to pattern recognition and dimensionality reduction (Jolliffe & Cadima, 2016 ▸). The process consists of a data decomposition in which samples, characterized by a dimensionality *p*, equal to the number of descriptor values (*e.g.* energies in XRF, 2θ angles in XRPD), are projected in a new space in which the directions of the new axes (named ‘principal components’, PCs) are defined by a linear combination of the starting variables (Jolliffe & Cadima, 2016 ▸). These PCs are generated by maximizing the explained variance, which means that they will be hierarchically generated depending on how much each PC describes the variance of the system (PC1 will have the maximum explained variance, PC2 will have less explained variance, and so on) (Jolliffe & Cadima, 2016 ▸; Guccione *et al.*, 2021 ▸). In a series of XRPD data sets obtained from a group of samples with different compositions, the main differences, *i.e.* the variance, are associated with the changes of the experimental intensities due to the different phase weight fractions in different samples.

MSA applied to X-ray measurements has started to develop in recent decades and is still a relatively novel field, as described in a recent review (Guccione *et al.*, 2021 ▸). PCA, in particular, has been applied to both single-crystal and powder X-ray diffraction for *in situ* experiments (Lopresti *et al.*, 2021 ▸; Conterosito *et al.*, 2020 ▸; Palin *et al.*, 2019 ▸; Matos *et al.*, 2007 ▸; Guccione *et al.*, 2018 ▸), and when combining different techniques such as XRPD and Raman spectroscopy (Urakawa *et al.*, 2011 ▸) or XRPD and pair distribution function (PDF)/UV–Vis (Caliandro, Altamura *et al.*, 2019 ▸; Caliandro, Toson *et al.*, 2019 ▸). Concerning XRF, the use of MSA is already a consolidated practice. In particular, methods such as partial least squares (Höskuldsson, 1988 ▸; Wold *et al.*, 2001 ▸) and principal component regression (Hotelling, 1957 ▸; Jolliffe, 1982 ▸) have been widely reported in the scientific literature (Grieken & Markowicz, 2001 ▸; Ghasemi *et al.*, 2013 ▸). MSA-based methods do not use crystal structure or other *a priori* known information but do use a probe-independent approach to tackle the same problem as the traditional methods, *e.g.* estimating scale factors between experimental XRPD and XRF intensities (typically the whole XRPD patterns and a sub-range of XRF spectra are used as input) and phase or element weight fraction in XRF [equation (2)] and XRPD [equations (6) and (7)], respectively. No specific equations are used in MSA, and each approach has specific data-analysis guidance criteria.

The multiple regression approach, fully described by Caliandro & Belviso (2014 ▸), is a whole pattern regression technique in which the experimental mixture profile 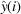 is fitted with a model *y*_mod_(*i*) in the form of 

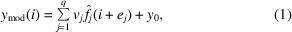

built using *q* pure phase profiles ( 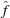 ). Mixture profiles are therefore treated as a linear combination of pure phase experimental profiles, and the parameters *v*_*j*_, *e*_*j*_ and *y*_0_, representing abundances of the *q* pure phases and the horizontal and vertical offsets of the profiles, respectively, are refined using the *MINUIT* libraries (James & Roos, 1997 ▸). This algorithm is implemented in *RootProf* and takes the name of MultiFit (Caliandro, 2020 ▸).

To prepare the data for PCA or regression procedures and overcoming the lack of equations, as in RR and FP methods for XRPD and XRF, respectively, it is often necessary to go through an experimental pattern pre-processing phase, which uses several mathematical tools (normalization, scaling, raising to a power, among the many possibilities) to improve the signal-to-noise ratio (Wehrens, 2011 ▸). This is a key step in the scale-factor estimation and weight or element fraction calculations, affecting the performances of all MSA methods. Pre-processing is based on mathematical treatments (Caliandro, 2020 ▸; Caliandro & Belviso, 2014 ▸) able to transform a raw experimental pattern into a pattern where the information needed for quantification is enhanced and background and biased intensities are suppressed. The typical example is the pre-processing of data sets showing PO, where the mathematical transformation suppresses the oriented peaks to overcome such bias. The used pre-processing approaches are described in Section 2.2, while their test, selection and optimization for the XRPD and XRF cases are described in Section 3.2.1. In this article, MSA was performed by using three different approaches:

(*a*) Supervised multiple regression analysis (SMRA), in which the scale factor for each phase composing the mixture is estimated by multivariate linear regression methods using pure phase patterns for fitting and standard mixtures with known composition for calibration and pre-process selection.

(*b*) Unsupervised multiple regression analysis (UMRA), in which the same regression methods as SMRA are exploited on samples, this time using pure phase patterns only. All the mixture patterns are used for quantification.

(*c*) Blind analysis (BA), in which pre-processed data are analysed by PCA without prior knowledge of mixture composition or pure phases. The guidance towards phase scales is given by the maximum data variance principle. The quantification is performed not by regression methods but by calculating the relative distances between the points in the PC space. All the patterns, including pure ones, are used for quantification.

### 1.3. Purpose of the work

Despite the fact that XRPD and XRF are often exploited alone or together, no systematic study focused on the performance of the MSA methods applied to XRPD and XRF methods of analysis in the full composition range is available. With the present article, we intend to fill the gap, assessing the performances of PCA, multiple regression and hybrid (PONKCS) approaches, in comparison with traditional methods (FP and Rietveld). XRPD and XRF data sets are analysed separately to assess the performances of the various methods and give recipes for the application of MSA methods to XRPD and XRF data. The goal is favouring the diffusion of multivariate approaches in all academic and industrial environments where solid materials are of interest and a large number of samples, in a wide range of compositions, must be analysed, thus making complex preparation procedures such as pearl fusion and milling impossible, or when the sample must be analysed in a nondestructive way. Determining the phase and element content in complex mixtures, such as the ones used for instance in brake pads, is a challenging task in quality control. Those mixtures are composed of reinforcing fibres, binders, fillers, lubricants and abrasives. Reinforcing can be carried out with ceramic materials such as potassium titanates; commonly used lubricants are graphite (C) and metalsulfide (*e.g.* MoS), and commonly used fillers are barite (BaSO_4_) or calcium carbonate (CaCO_3_), typically calcite. Quantifying the phase content in these mixtures with strong MA effects is very complex with XRPD and, due to the presence of graphite, it is also very complex from the XRF point of view.

Four sets of samples with PO and/or MA issues were prepared and analysed by XRF and XRPD. Substances for the mixtures were selected following different criteria in order to simulate real examples of complex mixtures: (i) presence of both organic and inorganic substances difficult to quantify by traditional methods due to PO and/or MA phenomena; (ii) non- or low-toxicity of the components so that they could be easily handled; (iii) non-reactivity in mixture in standard conditions; and (iv) wide use in general industry. In all the mixtures, the two heavily absorbing phases are bismite (Bi2O_3_) and barite (BaSO_4_) with LACs of 1978 and 924 cm^−1^, respectively, using a Cu X-ray tube. A third lighter phase is added to these two heavy phases to obtain four ternary mixtures: sieved graphite (LAC of 10.18 cm^−1^) in specimen D1; oriented graphite in specimen D2; zinc acetate, an organic sample but with Zn *K*α recorded in XRF data (LAC of 40.97 cm^−1^), in specimen D3; and urea (LAC of 9.91 cm^−1^) in specimen D4. The space represented by the mixture weight fractions, *i.e.* the corresponding ternary phase diagram, is commonly defined as the ‘experimental domain’ (Cornell, 2011 ▸).

The most efficient way to explore an experimental domain is through the use of the design of experiments (DoE) approach (Box *et al.*, 1978 ▸; Cox & Reid, 2000 ▸; Cornell, 2011 ▸). The DoE approach consists of a set of mathematical tools allowing one to plan experiments to extract the maximum possible amount of information contained in the experimental domain with the least number of experiments. A DoE approach was used to prepare the mixture samples, to cover all the space represented by each possible combination of the phases’ weight fractions in a controlled and efficient way as described in detail elsewhere (Mangolini *et al.*, 2021 ▸). Each set of samples was then analysed by XRPD and XRF to produce four data sets. The obtained XRPD/XRF data belong to a collection of data stored in a online repository that we recently created (https://doi.org/10.17632/js2nzwf5md.2). The database is open to new contributions, with the aim of creating a large data set for testing and calibrating XRPD and XRF techniques. The features and instructions to exploit the current data or for adding new data are given in a dedicated publication (Mangolini *et al.*, 2021 ▸).

In the present article, these data are analysed both by the traditional approaches and by the above-described multivariate analysis approaches (SMRA, UMRA and BA). A detailed description of the pre-processing optimization and selection is given, being the key step to obtaining the best QPA performances among all the adopted approaches. The goal of these approaches is managing, with a reasonable precision, complex mixtures to allow fast (and in principle automatic) processing and analysis of a large number of samples. Moreover, the hybrid method PONKCS was tested to compare its performance with respect to RR and the pure multivariate approach. PONKCS was originally developed to refine with a Rietveld-like approach phases whose structure is either not known or only partially known. In this work, we exploit PONKCS to obtain better estimates for light phases in samples affected by MA, even if their crystal structure is known. Moreover, only one of the four data sets will be analysed by XRF (*i.e.* data set D3) because both graphite and urea lack elements that give an XRF signal detectable by the used low-power benchtop instrument.

## 2. Materials and methods

### 2.1. Data collection

Sample preparation, morphological characterization, instrumentation and data collection are described in a dedicated publication (Mangolini *et al.*, 2021 ▸). Ternary mixtures were prepared by a DoE (Cornell, 2011 ▸; Cox & Reid, 2000 ▸) approach to properly sample their full compositional range. All the data sets have been made available in an open online database (Mangolini *et al.*, 2021 ▸). Table 1 ▸ shows the main features for each sample.

### 2.2. Software

FP analysis of XRF data was carried out by the proprietary software installed in the XRF instrument (Rigaku, 2012 ▸). This being a benchtop/portable instrument with a low-power (4 W) X-ray tube, the *K* line for carbon cannot be observed. XRPD data were analysed by the traditional RR approach using *TOPAS-Academic* (V5) (Coelho, 2018 ▸, 2020 ▸). A whole profile regression (using the MultiFit algorithm) and PCA-assisted quantitative analysis were performed by using *RootProf* version 14 (Caliandro & Belviso, 2014 ▸). This software includes different pre-processing options organized into four classes (named levels), and one action for each level is executed on raw data one after the other. The levels of the modification functions are profile modifications (level 1), rescaling (level 2), background subtraction (level 3) and filtering (level 4) (Caliandro & Belviso, 2014 ▸), whose use is documented in a dedicated web page with dedicated tutorials for its efficient learning and usage (Caliandro, 2020 ▸).

These pre-processing steps have the scope of transforming raw data into modified data where background and bias are suppressed and relevant information (phase or element amounts in XRPD or XRF, respectively) is dominant. Some useful and widely used raw-data pre-processings are still not included in *RootProf* (Savitzky–Golay filtering and autoscaling), and were thus performed by using R base version 4.1.0 (R Core Team, 2013 ▸) and the *prospectr* package version 0.2.1 (Stevens & Ramirez-Lopez, 2021 ▸). The hybrid approach (exploiting, at the same time, Rietveld refinement and a MultiFit-like approach using the pure phase intensity information) named PONKCS (Scarlett & Madsen, 2006 ▸), as implemented in *TOPAS-Academic* (V5) (Coelho, 2018 ▸, 2020 ▸), was used for a wide exploration of possible analytical solutions.

## 3. Results

Eight data sets were built, collecting XRPD and XRF data on four mixtures belonging to a ternary experimental domain, whose features, as summarized in Table 1 ▸, were prepared in the whole concentration range with the aid of an augmented simplex-centroid DoE (Cornell, 2011 ▸), as introduced in Section 2. The analysis of XRPD and XRF was carried out comparing, in both cases, traditional (Rietveld for XRPD and FP for XRF) and multivariate methods (SMRA, UMRA and BA). Moreover, XRPD data were analysed by PONKCS, as implemented in *TOPAS* V5 (Coelho, 2020 ▸).

### 3.1. Traditional methods

#### 3.1.1. Rietveld analysis

**Figure 1:**
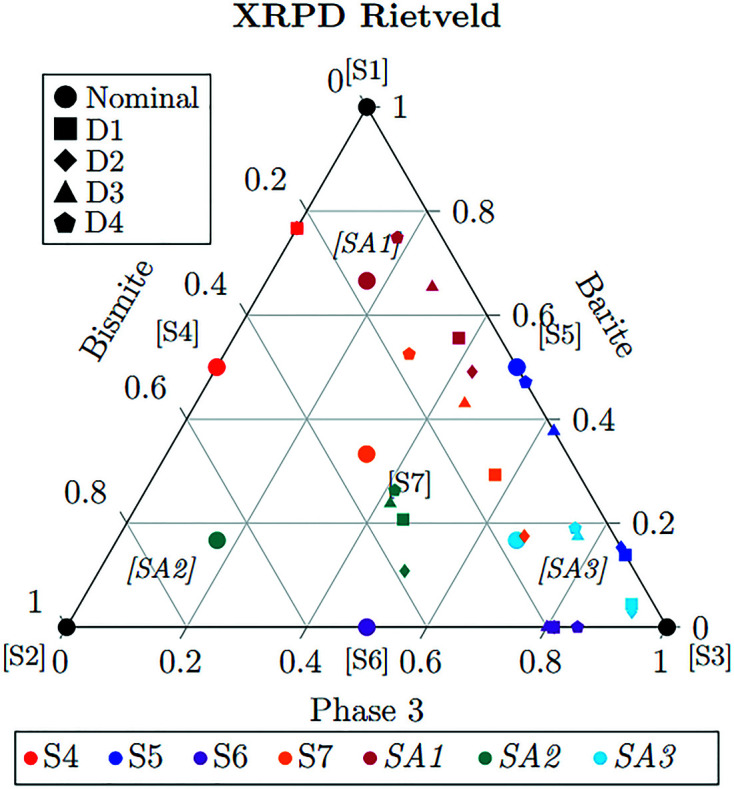
Results of the XRPD Rietveld analysis reported on the ternary graph representing the mixtures’ experimental domain; S4–S7 are ternary and binary mixtures of the simplex DoE, while SA1–SA3 are the augmented simplex samples, highlighted in italic. Phase 3 is the lighter phase: graphite, oriented graphite, zinc acetate and urea in data sets D1, D2, D3 and D4, respectively.

The four XRPD data sets were refined first by a normal RR with a one-direction March–Dollase correction parameter for PO for graphite. The RR data (Fig. 1 ▸) are represented with the following labelling scheme: Each symbol is associated with one out of the four data sets. Each composition is related to a specific colour: S4, S5 and S6 are the binary mixtures with 50% in weight of each component; S7 is the 33% equivalent weight ternary mixture; and SA1, SA2 and SA3 are the augmented mixtures (66.6%, 16.7%, 16.7%), (16.7%, 66.6%, 16.7%) and (16.7%, 16.7%, 66.6%), respectively. With this scheme, the aggregation of symbols of the same colour close to the circles (representing the expected nominal values) indicates small deviations from the nominal value. As expected, the symbols in Fig. 1 ▸ are rather dispersed, highlighting large deviations in the weight fractions estimated by the standard RR approach. The Rietveld profile fitting reaches a satisfactory agreement factor (*R*_wp_ < 17); also, when strong MA is present, unless the analyst knows the actual sample composition, there is no evidence from the RR results that something should be improved or changed (Fig. S1 of the supporting information).

In general, because of MA, high-absorbing barite and bismite are underestimated and the lighter Phase 3 is overestimated in every data set. In sample S4, common to all data sets, barite–bismite 50:50 MA is present and the barite content is overestimated at 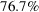 . Deviations due to strong MA are highlighted for samples S5 of data sets D1 and D2, composed of 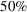 barite and 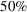 graphite as the lighter phase, and for samples S6 of all data sets, composed of bismite and a lighter phase at 50% in weight. This behaviour affects the deviations observed in the RR fit from the expected values of the ternary mixture. The mean deviation computed on samples S7, SA1, SA2 and SA3 for D1 is due to an underestimation for barite of 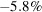 , an underestimation for bismite of 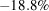 and an overestimation of 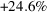 for graphite. Similar behaviour is seen in data set D2. In the case of D3, where zinc acetate replaces graphite, the mean deviation computed on the samples S7, SA1, SA2 and SA3 is lower and bismite is underestimated by 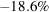 , and barite and zinc acetate are overestimated by 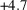 and 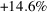 , respectively. A similar behaviour is seen in data set D4, where urea is present.

**Figure 2:**
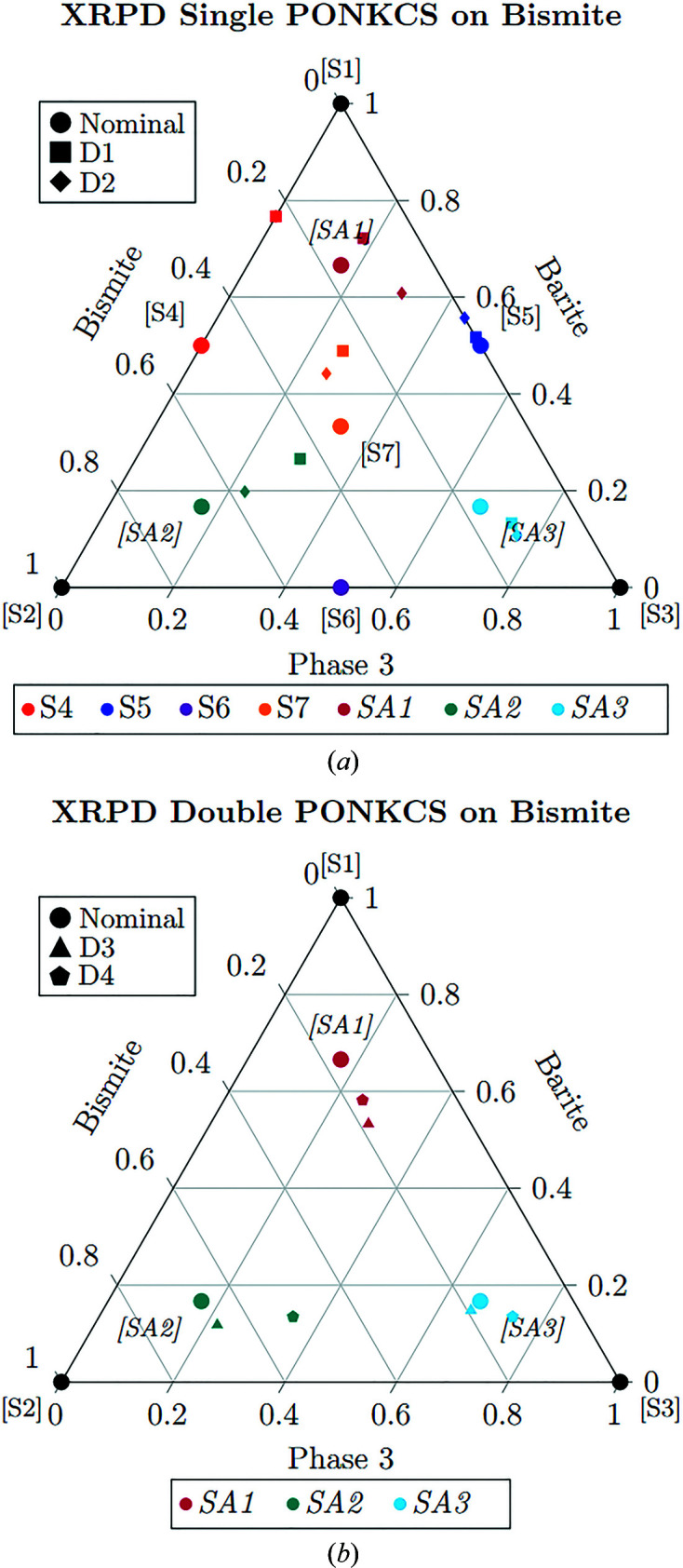
Results of the XRPD PONKCS analysis reported on the ternary graph representing the mixtures’ experimental domain. Labelling and colour scheme as in Fig. 1 ▸.

The PONKCS approach (Appendix *A*3[App appa]) is exploited to ‘calibrate’ and try to properly manage the MA effect, still by using the RR approach. As seen in Fig. 2 ▸, the values are much less dispersed compared with the classical RR case (Fig. 1 ▸). In the case of data sets D1 and D2, the best approach is the single PONKCS (see Appendix *A*3[App appa] for a detailed definition of single and double PONKCS) calibrated on sample S6, with the under- and overestimation of heavier and lighter phases much more limited than for RR. Instead, for data sets D3 and D4, the best approach is the double PONKCS calibrated with respect to the bismite content on sample S7. In the case of single PONKCS for data sets D1 and D2, the mean deviation from the expected values decreases from 17.1 and 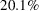 to 8.7 and 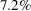 , respectively. In the case of double PONKCS for data sets D3 and D4, the mean deviation decreases from 12.6 and 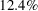 to 4.7 and 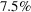 , respectively. The squared sum of the residuals (SSR) of estimated phase abundances from the RR and PONKCS with respect to the actual value is reported in Table 2 ▸ (taking the sum of the phases as equal to 1).

#### 3.1.2. XRF FPs result and measurement conditions

For these kinds of mixtures, with phases with very different absorption coefficients, XRF results are dependent on measurement conditions. Measuring at 50 kV with an Ag X-ray tube and an Ag filter placed between the tube and the sample allows a smooth background at low energies, cutting the *L*α of the Ag tube, but the NexQC low-power X-ray tube, as a portable instrument, is not able to excite sufficiently and record intensities of the *K*α of carbon, even in the presence of a helium purge. With the classical FP quantification approach, analysing the *L*α emission line of bismuth and the *K*α line of barium, it is possible to quantify only the relative amount of barium and bismuth in the mixture without the contribution of the lighter phase. The presence of the lighter phase, graphite or urea, does not affect the intensities of Ba *L*α and Bi *K*α. Those intensities are independent of the lighter-phase concentration (*e.g.* in mixtures S4, S7 and SA3 where the integrated intensities of the fluorescence emission lines *L*α for bismuth and *K*α for barium have a constant value). The case of data set D3 with zinc acetate is more straightforward due to the presence of the Zn *K*α emission line, for both the classical FP method and the MSA approach.

### 3.2. Multivariate analysis of XRPD/XRF data

#### 3.2.1. Pre-process selection

The multivariate approach has the great advantage of being probe independent, so it can be applied in the same way to XRPD and XRF data, and it does not require known crystal structures or other *a priori* information. Moreover, no relations, such as those in equations (4)–(7 [Disp-formula fd6] [Disp-formula fd7]) (XRPD) or equation (2) (XRF), are assumed to relate experimental intensities and phase or element fractions. Therefore, the system can be analysed in an unbiased way, driven by the specific features of the experimental profiles. As a drawback, the lack of information about scale factors and the absorption coefficients of each component of the mixture must be compensated by the use of other guiding principles. On the one hand, each approach has an intrinsic principle underlying the multivariate analysis, *e.g.* variance in PCA-based BA or the minimization of the SSR towards pure phase patterns in multiple regression (SMRA and UMRA), as described in Section 1.2. On the other hand, the power and flexibility of the multivariate method rely on the almost infinite combination of mathematical tools used to transform raw patterns to suppress noise and bias and enhance the information useful for quantification. These data pre-processings might drastically transform the pattern, but this is the way to obtain good quantitative results. In this mandatory preliminary analysis named ‘pre-process selection’ (see Section 1.2), before investigating unknown samples, the effects of many parameters (such as the 2θ data range, re-scaling of intensities and re-sampling of the profiles by a smoothing algorithm) must be evaluated. In this section, the adopted approach for the chosen very difficult case study is presented, while in Section 4 a guide for the best approach depending on sample features and experimental needs is given. In fact, when PO and MA are present and the whole experimental domain is studied, the difficulty is at its maximum, and this preliminary phase can be very time and resource demanding, requiring a suitable training data set of known samples. Pre-process selection is performed by *RootProf* in an automatic way through its calibration process in supervised analysis (Caliandro & Belviso, 2014 ▸).

For this study, additional, still not implemented within *RootProf*, pre-processes were tested (Savitzky–Golay filtering and mathematical derivative) using the R framework (R Core Team, 2013 ▸). This external pre-process optimization followed an experimental factor design approach of 2^5^ (Box *et al.*, 1978 ▸; Cox & Reid, 2000 ▸), where the pre-processing parameters smoothing window, derivative order and autoscaling were combined with the 2θ range of the pattern and the number of skipped data in the *RootProf* calibration process. The SSR of estimated phase abundances with respect to the measured one was used to identify the best combination of pre-processing parameters, and the optimization was performed on each XRPD and XRF data set separately. For convenience, only the best pre-process combinations have been reported in Table 3 ▸. The details about all remaining pre-process combinations can be found in the supporting information. Concerning XRPD profiles, Table 3 ▸ shows that the best results are obtained by analysing their full range and not subranges containing only the highest-intensity peaks. However, the *RootProf* internal pre-process showed the existence of a better option, which occurs more frequently among the XRPD data sets: *L*_1_ = 3 (logarithm in base 10 of the pattern intensities), *L*_2_ = 2 (normalization of the subtended area to 1), *L*_3_ = 0 (no background subtraction) and *L*_4_ = 3 (PC filtering). The powering of the pattern intensities to 4/5 (*L*_1_ = 4) seems to be another good alternative for profile modifications, while *L*_4_ = 4 is a variant of PC filtering *L*_4_ = 3. The autoscaling performed by R did not give any valuable result in the presence of PO and MA. The same procedure was repeated without taking into account the conditions found to be more unfavourable, such as the autoscaling, the first-order derivative and the 2θ range reduction.

For XRF data, for data set D3 with zinc acetate, the internal *RootProf* pre-process confirms that the best performing combination has *L*_4_ = 3 (PCA filtering). Excluding the tail at the beginning and end of the XRF spectra, where values are going noisily to zero, is also crucial. Having determined the best pre-processing options, we present the performance of the multivariate approach on both XRPD and XRF data sets in the following section, by using supervised and unsupervised QPA and a completely blind analysis, where no information (not even the pure phases) is supplied to the software.

#### 3.2.2. Supervised quantitative analysis

**Figure 3:**
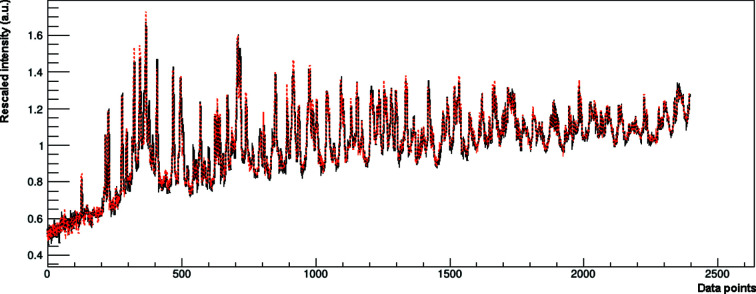
A fit (red dashed line) produced by *RootProf* using the MultiFit algorithm during SMRA on sample SA1 of data set D1. The pattern (black continuous line) has been pre-processed as reported in Table 3 ▸.

**Figure 4:**
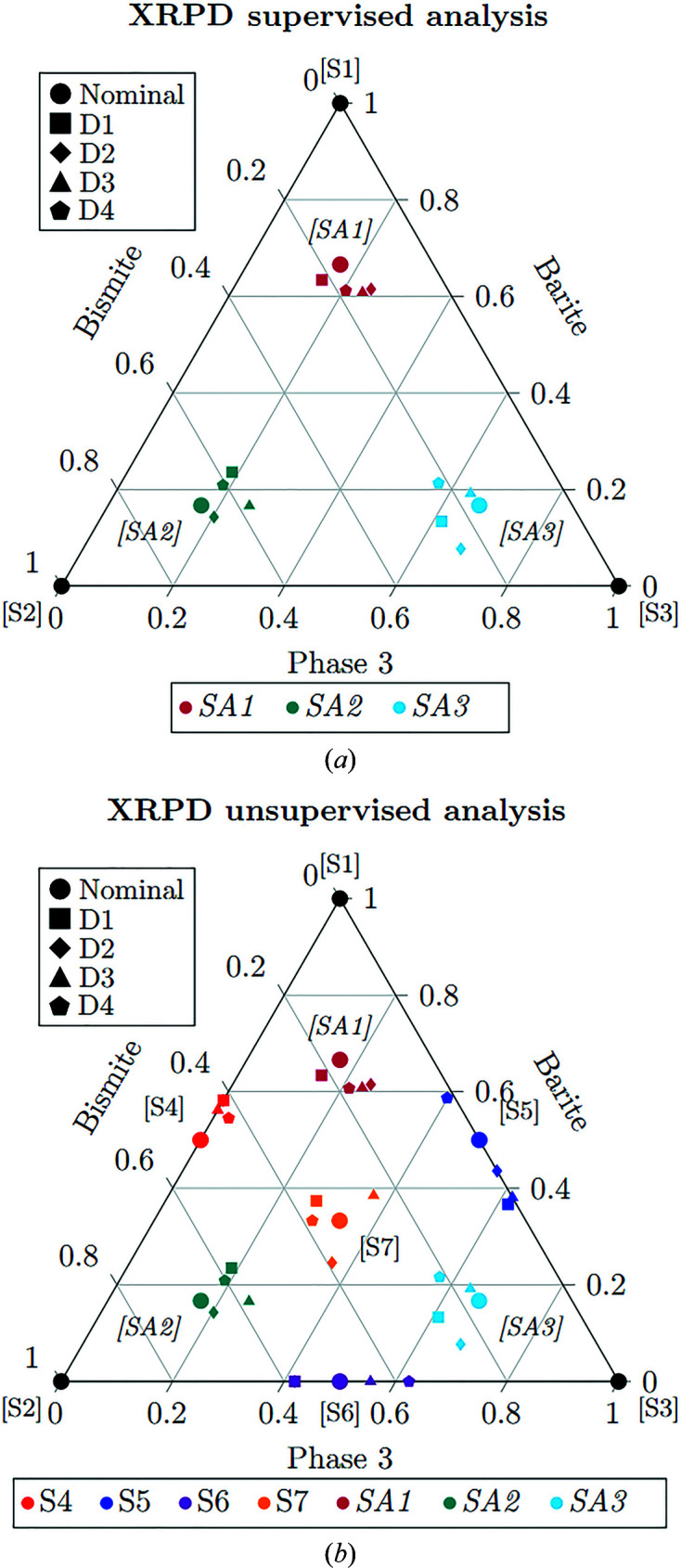
Results of the (*a*) SMRA and (*b*) UMRA performed on XRPD data, reported on the ternary graph representing the mixtures’ experimental domain. Labelling and colour scheme as in Fig. 1 ▸.

SMRA was performed using the three pure phases and the four other simplex mixtures to calibrate the model, while the augmented experiments SA1, SA2 and SA3 were used as unknown samples to test the procedure. XRPD data sets were analysed using the best pre-processes obtained by the selection procedure described in the previous section. In Fig. 3 ▸, an example of the performance of the MultiFit procedure on data set D1 is given. In this figure, the goodness of the fit performed by *RootProf* is evident, like the RR reported in the supporting information (see Fig. S1). The results of the data analysis of the four data sets are reported in Table S1 of the supporting information and Fig. 4 ▸. The data are closer to the expected value, even in comparison with the PONKCS calibrated approach (Fig. 2 ▸).

Data set D1 has uncertainties on the estimations of up to 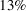 , as can be seen for sample S5. In data set D2, surprisingly since the average particle size of graphite is larger than that in data set D1, individual results are generally more precise than those obtained for data set D1, and the SSR is reduced. In these data sets showing PO and MA, SMRA appears to be rather more robust than RR and PONKCS. In fact, significantly lower SSR (see Table 2 ▸) values are observed for the results obtained by multivariate analysis compared with the best performing PONKCS (SSR_D1–SMRA_ = 0.027, SSR_D1–PONKCS_ = 0.0899). Data sets D3 and D4 show errors similar to the first two data sets, always below 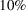 in the estimates. The error distributions are always normal and zero-centred, a sign that systematic errors are absent, or very limited (analyses of residuals are reported in the supporting information), in contrast to PONKCS and RR. XRF data set D3, the only data set with the presence of an XRF active element in all three species, was analysed using the best pre-processes obtained by the selection procedure described in the previous section and using the XRF spectra obtained at 50 kV. As expected, the FP algorithm performs better in the SA2 sample case where the lighter phase zinc acetate is the minority phase. In the other two cases, the results of the SMRA approach are comparable to those of the FP method. Globally, very similar SSRs are observed, with a value of 0.104 for the FP method and 0.111 for SMRA. This approach represents typical real-world use of the regression method, especially in complex cases and when errors must be minimized. After the pre-process selection and optimization have been performed on a well defined series of samples, *e.g.* clinker in a cement company or graphite in a lubricant plant, the SMRA method can also be implemented for routine analysis in a fully automatic approach.

#### 3.2.3. Unsupervised quantitative analysis

UMRA was performed on XRPD and XRF data by supplying to the software information regarding the pure phases, while each other mixture was used for testing the fitting method. A pre-process combination was applied, exploiting general-use recipes for samples without particular critical issues (Caliandro, 2020 ▸), or using indications by a previous SMRA calibration (Table 3 ▸), as carried out in the present article because of the presence of MA and PO. Since UMRA is a standardless method, it can be applied successfully when a strong correlation between the scale parameters of the experimental profiles after the pre-process and the quantities in the mixtures is present, as demonstrated in the previous section. In this case study, this was found to be true for XRPD data but not for XRF data, and thus XRF data are not reported for UMRA in the present work. The results of the quantification are reported in Table S2. For data sets D1, D2 and D3, the estimations are very similar to those obtained by performing SMRA, and the quantitative information can be extracted from the data, since the pre-processing procedure is already known after the calibration by SMRA in the previous section. Similar pre-process options were found for all data sets, suggesting a rather general approach, as debated in detail in Section 4. The range of the errors spans from 0 to 12.4%, which is still a good value for these kinds of samples, and it represents the best result among the three different QPA approaches. As for supervised QPA, the residual analysis does not show any easily recognizable trend, and the error distribution is normal and zero-centred. Unsupervised QPA of XRF data was not reported due to the large errors (up to 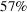 error in quantification of zinc acetate).

#### 3.2.4. Blind analysis

BA was run on each data set to test the limits of the minimum required knowledge needed by the MSA to perform a very fast semi-quantitative analysis without any *a priori* information. Differently from SMRA and UMRA, no compositional or pure pattern profile information was given as input to the *RootProf* software. BA relies only on the explained variance extracted by PCA and will be based on the relative distances between the samples projected in the PC spaces.

**Figure 5:**
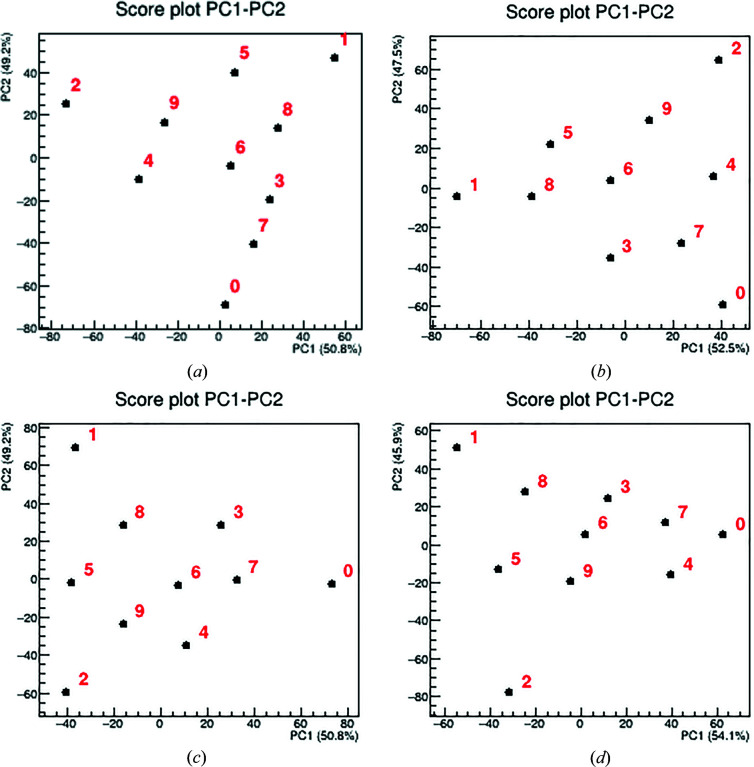
The results of the BA are reported in the form of score plots for each data set: (*a*) D1, (*b*) D2 , (*c*) D3 and (*d*) D4. The numbers represent the positions of the samples in Table S3 (0 is Ba, 9 is SA3).

The results of BA are reported in the form of a score plot for each data set (Fig. 5 ▸) and as numerical values (Table S3). In the former, the points represent each experiment of the simplex projected in the space described by the first two PCs. In every score plot, the disposition of the points resembles the one designed by the simplex-centroid augmented experimental design. In this case, the estimation of the mixture’s content can be performed either by a geometrical approach, based on relative distances of each point from the vertices (which can be identified as the pure phases), or by the application of the transformation proposed by Cornell (2011 ▸) in order to change from the Cartesian coordinate system into the barycentric coordinate system of a ternary plot. In this work, the second option was pursued, with a custom code programmed in R language that projects the sample points on a ternary graph space. Good accuracy in the estimations can be achieved (SSR down to 0.078 for data set D2), but when very evident MA or PO issues are present, large errors in the phase quantification can be found using BA, *e.g.* in mixture S4 of data set D4, urea is underestimated by 26.4%. However, if an accurate quantification is not required, this is a fast method for efficient semi-quantitative analysis of a set of unknown samples of similar origin without *a priori* knowledge of any kind. This approach was not used to analyse XRF data, as the results of UMRA already had large errors and BA is, of course, even worse.

## 4. Discussion

**Figure 6:**
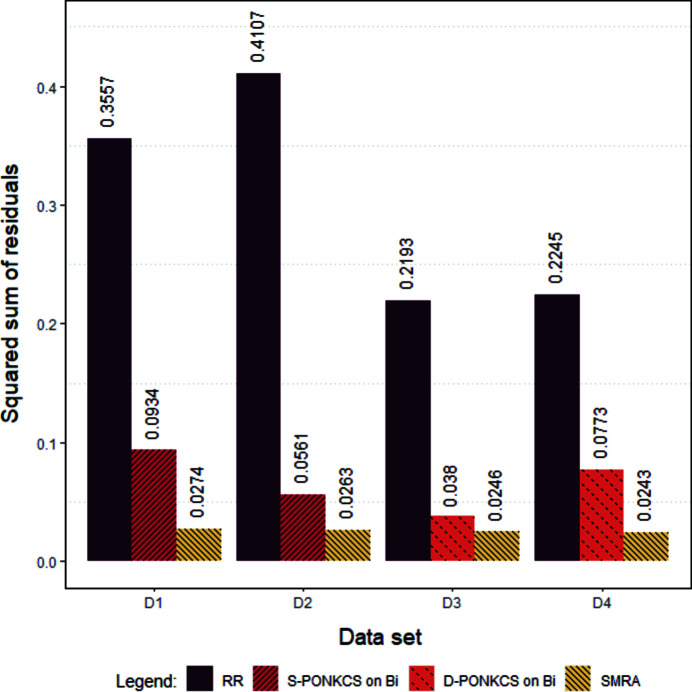
The exploited quantification methods’ performances are compared using the resulting SSR obtained on each data set.

The analysis and comparison of the results of the various approaches on both XRPD and XRF data sets allowed us to understand the potentialities and limitations of each method. Moreover, it is possible to provide users with guidelines for choosing the more suitable approach, taking into account both sample features and expected analytical precision. To allow such a comparison, a selection of SSR values has been reported in the bar graph of Fig. 6 ▸ to better illustrate the difference in the accuracy of the different quantification methods. All the corresponding numerical results can be found in Table 2 ▸, and the corresponding original quantifications for each technique and data set can be found in Tables S1–S7 of the supporting information.

The multivariate methods, in some cases, outperformed the traditional methods for XRPD data, while only SMRA was comparable to classical FP results for XRF data (Table S7). For this reason, only the XRPD data-analysis approach is discussed in detail. As is well known, RR is the method of choice if no standard is available, PO and MA are limited, and all the crystal structures are known. Similarly, the PONKCS approach is the option to choose when some crystal structures are not known but pure phase patterns are available, and SMRA is not suitable in the absence of the standard for the calibration. The results of XRPD phase quantification from classical RR in the presence of a strong MA effect showed large errors, as expected. The PONKCS approach was successfully applied, depending on the data set features in its single and double versions (as described in Appendix *A* [App appa]), with much lower errors than RR. Interestingly, the multivariate approach and, in particular, its supervised version (SMRA) outperformed both PONKCS and RR for XRPD data and obtained similar results to the FP method for XRF data. Therefore, if both pure phases and standard mixtures are available, the SMRA approach is the only approach able to quantify the relative amount of each phase with minimal errors, if MA and PO are present. In these difficult case studies, at first the Savitzky–Golay filtering was effective in reducing the noise in the data. This result is demonstrated by comparing the errors on the phase estimation (SSR) during the calibration of the pre-process (see the tables in the supporting information .xlsx file). Moreover, key pre-processing options for QPA turned out to be the PCA filtering coupled to logarithmic scaling (preprocess 3 2 0 3 in *RootProf*) or raising to the power 0.8 (preprocess 4 2 0 3), which were able to suppress the intensity bias in the presence of MA and/or PO.

UMRA can be a faster alternative to SMRA when an efficient pre-process combination is already available, using standard recipes (Caliandro, 2020 ▸) or from a previous SMRA on similar samples, as in the case of PO and MA (Table 3 ▸), and a fine control of errors is not needed. In fact, the lack of calibration standards while calculating the model on unknown samples makes the methods less time consuming but also less robust and with larger expected errors. This approach represents the condition in which a strong correlation between the scale parameter of the profile after the pre-process and the quantities of the single components in the mixture exists. It can be used when a high accuracy in the results is not required or when it is impossible, for any reason, to produce standards at known quantities. BA is the best solution to obtain semi-quantitative analysis of XRPD data and is a fast approach to analyse many samples whose features are already known in a minimal amount of time. Moreover, BA is the only possibility when no structural knowledge or pure phase profiles are available. Given the potentialities of these multivariate approaches, in terms of outperforming RR and PONKCS in both precision and efficiency of the analysis especially when managing very large data sets, a discussion on the selection of the data-analysis method and the pre-processing combination is mandatory.

**Figure 7:**
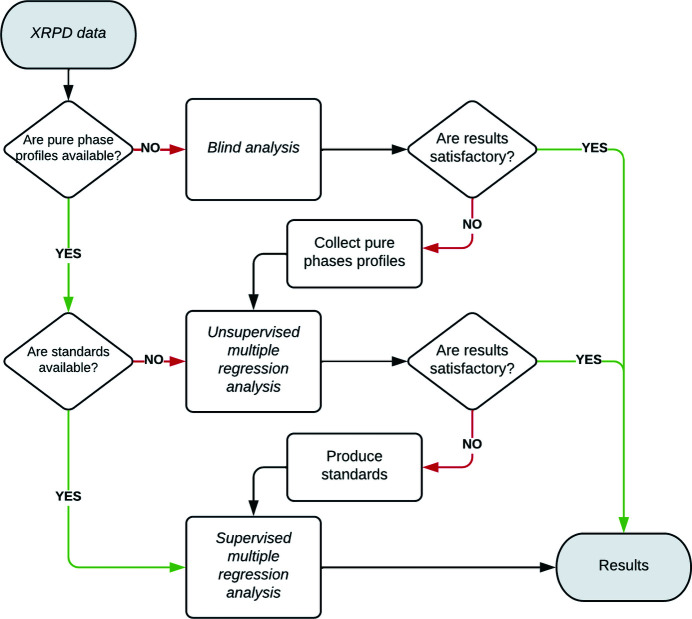
A summary flowchart of the different ways in which quantitative analysis on XRPD data can be approached, as proposed in the present work.

In Fig. 7 ▸, a possible flowchart for the application of multivariate methods, based on the available data, is given. Of course, as discussed before, the aim of the analysis (precision, number of samples, automation) should also be taken into account. The more rigorous and suggested approach is replicating the work carried out in the present case, *i.e.* SMRA after measuring sample, standard and pure phase profiles with the selection of the best pre-process (bottom of Fig. 7 ▸), as described in Section 3.2.1. A number of known samples (three to five or more depending on the complexity of the case study and the size of the experimental domain) are necessary to carry out a supervised analysis (SMRA) to assess the best pre-process combination, with the exact number of standards depending on sample complexity and accuracy expectations. In this case, results with maximum precision and accurate control of the errors, without external validation, can be obtained. However, the SMRA approach with a full calibration can be unsuitable because it is not feasible (pure phases and/or standards not available), not efficient (many samples to be analysed) or not necessary (high precision not needed).

In one of these cases, the flowchart of Fig. 7 ▸ can be used from top to bottom. A PCA-based BA approach can be adopted, by using a suitable pre-process as discussed in Section 1.2, to efficiently identify the number of possible phases, and their estimated quantities and the results can be validated against complementary information (elemental analysis, sample history, microscopy evaluation). Moreover, samples can be classified by BA into families, highlighting similarities and differences. Then, if quantitative information is needed and pure phase profiles have been measured, UMRA, with the same pre-process as selected in the previous BA, can be executed. In this case, the results need an external validation like BA, but the average errors are in general much smaller, as discussed in Section 3. Finally, to avoid external validation, the user must adopt SMRA, typically combined with pre-process optimization for each single case study or family of samples. With Fig. 7 ▸ in mind, each user can find the most suitable and efficient approach for their specific experimental needs and sample number and complexity. The *RootProf* tutorial page (Caliandro, 2020 ▸), or any other software suitable for MSA, contains all the technical information to start and carry out the analyses.

## 5. Conclusions

Four XRPD and four XRF data sets were produced, using a DoE approach, by preparing ternary mixtures obtained by combining low-density phases (organic as graphite and urea, and hybrid as zinc acetate) with high-density materials (barite and bismite). The purpose was exploring and comparing, in a systematic and standardized way, data-analysis approaches for quantitative phase evaluation in real-world-like situations, as discussed in the *Introduction* [Sec sec1]. Simplex-centroid augmented experiment design was used to explore the whole experimental domain, and the obtained data sets were deposited in an open database that is described in a separate publication (Mangolini *et al.*, 2021 ▸).

For XRF, only SMRA gave results comparable to traditional FP methods. Conversely, XRPD patterns showing MA and/or PO were hard or impossible to refine by traditional Rietveld methods without using the calibrated PONKCS approach. Both supervised and unsupervised multiple regression analyses performed better than the hybrid PONKCS approach and significantly outperformed traditional RR, whose average errors (SSR in Table 2 ▸) were much larger. Also BA by PCA, recently introduced by some of us in the XRPD field (Guccione *et al.*, 2021 ▸), gave good estimations (SSR down to 0.078). The good performances of multivariate methods were obtained on samples recalcitrant to traditional approaches, without costly and time-consuming sample preparation, avoiding milling, and thus simulating real-world usage with the analysis of many (hundreds per day) samples. The possibility of automation and self-learning of multivariate approaches offers new possibilities in quality-control procedures when dealing with complex solid mixtures that are hard or impossible to manage with the traditional Rietveld method. Finally, to help widen the use of multivariate methods in XRPD data analysis, a guide for the choice of the best approach (see Fig. 7 ▸ and its discussion) and indications about pre-process selection (see Section 1.2) have been given, depending on both the user’s needs and goals and sample features. 

## Supplementary Material

Supporting figure and tables. DOI: 10.1107/S1600576722004708/nb5320sup1.pdf

Quantification tables and preprocess calibration. DOI: 10.1107/S1600576722004708/nb5320sup2.xlsx

## Figures and Tables

**Table 1:** A summary of the characteristics of each sample analysed by XRPD and XRF More details on the characteristics of the samples are given by Mangolini *et al.* (2021 ▸).

Data set	Phases	A brief description of the mixture
D1	BaSO_4_, Bi_2_O_3_, sieved C	Graphite has an average particle diameter of 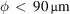 to introduce moderate PO effects. There are large differences in density of the three phases for an MA effect. There is absence of characteristic XRF signal for graphite.
D2	BaSO_4_, Bi_2_O_3_, mixed C	Same as sample D1 but this time graphite has a 30% in weight content with average particle diameter larger than 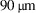 with pronounced PO effects.
D3	BaSO_4_, Bi_2_O_3_, ZnC_4_H_6_O_4_	All phases have an XRF signal. Zinc acetate introduces moderate PO and has a lower density than graphite, enhancing MA effects. Zinc acetate also has a larger unit cell, to increase peak superposition in XRPD.
D4	BaSO_4_, Bi_2_O_3_, CH_4_N_2_O	Absence of XRF signal, slight PO effect. Urea presents larger average particle size and has a lower density than zinc acetate, with increased MA effects.

**Table 2:** To make comparable the performances of the exploited methods, the differences between the expected values and the predicted values for each mixture belonging to each data set were calculated Overall performances were expressed as the SSR, commonly used to evaluate the agreement degree of the regression models.

	Data set D1		Data set D2		Data set D3		Data set D4	
Method	SSR_SA1–SA3_	SSR_TOT_	SSR_SA1–SA3_	SSR_TOT_	SSR_SA1–SA3_	SSR_TOT_	SSR_SA1–SA3_	SSR_TOT_
Rietveld	0.3557	1.046	0.4107	1.1642	0.2193	0.6393	0.2245	0.6798
Single PONKCS on barite	0.0899	–	0.1176	–	0.0741	–	0.1885	–
Single PONKCS on bismite	0.0934	–	0.0561	–	0.1831	–	0.5672	–
Double PONKCS on barite	0.1068	–	0.1109	–	0.0644	–	0.1613	–
Double PONKCS on bismite	0.1326	–	0.1787	–	0.038	–	0.0773	–
SMRA	0.0274	–	0.0263	–	0.0246	–	0.0243	–
UMRA	0.0292	0.0938	0.0263	0.0727	0.0246	0.0787	0.0259	0.0877
BA	0.0788	0.1678†	0.0161	0.0781†	0.0599	0.1129†	0.1528	0.3514†

† SSR_TOT_ for BA was calculated without including pure phases.

**Table:** XRPD data.

	External pre-process			*RootProf* setup		Results	
Run	Smoothing window	Derivative order	Autoscaling	2θ range	Skipdata	Best pre-process	SSR
D1	5	0	No	10–120	3	3 2 0 3	0.027
D2	5	0	No	10–120	3	3 2 0 3	0.026
D3	5	0	No	10–120	3	4 2 0 3	0.025
D4	9	0	No	10–120	5	4 2 0 4	0.24

**Table:** XRF data.

	External pre-process			*RootProf* setup		Results	
Run	Smoothing window	Derivative order	Autoscaling	Energy range	Skipdata	Best pre-process	SSR
D3	0	0	No	1.8–16.4	3	5 0 0 3	0.058

## Appendix A Physico-mathematical background on traditional data-analysis approaches

In traditional XRPD and XRF data analysis, analytical relations between single-peak or whole profile intensities are related to the weight fraction of a phase (XRPD) or the concentration of an element (XRF). The precision in the quantification relies on the adherence between used models and measured intensities. Traditional approaches to XRPD and XRF data analyses are well established; therefore, only their main equations are recalled in the following subsections to underline how weight fraction is related to experimental variables. Conversely, a detailed background is given for the multivariate methods in Section 1.2 to highlight similarities to and differences from the traditional approaches and to help the reader in understanding and exploiting the proposed MA methods.

### A1. FPs in XRF data analysis

The FP approach applied to XRF data allows one to obtain quantitative elemental results by using equations borrowed from the theory of X-ray interaction with matter (Criss & Birks, 1968 ▸; Grieken & Markowicz, 2001 ▸). It represents a way to determine approximate concentration results without the need for empirical calibration (which is costly and time consuming, requiring known assayed standards, and typically valid in relatively small concentration ranges). FP equations model the background, automatically deconvolute elemental-peak overlap, estimate elemental-peak areas and model X-ray absorption/enhancement effects by generating theoretical alpha corrections (Rigaku, 2012 ▸). The calculation of sample compositions is based on the general relationship between the concentration of the analyte *i* (*C*_*i*_) and its measured net intensity (*I*_*i*_), which is expressed by a very simple equation (Rousseau, 2006 ▸, 1984*a* ▸,*b* ▸):

where *k*_*i*_ is the calibration constant for the *i*th analyte, *I*_*i*_ is its measured intensity and *M*_*is*_ is the correction of the ME of the sample *s* on the *i*th analyte. The determination of the parameter *M*_*is*_ is the critical point. Its general applicability to, in principle, any sample represents a huge advantage, but the ME (and then the quantification error) can limit the reliability of the result. Different algorithms are used in modern manufacturers’ software and each will give, in principle, reliable results (Willis & Lachance, 2004 ▸).

### A2. RR in XRPD data analysis

RR is a method that was suggested by H. M. Rietveld between 1967 and 1969 for the analysis of powder diffraction data. It is a least-squares whole-pattern refinement that minimizes the differences between the experimental pattern and the calculated one, modelling the contributions coming from instrumentation and from each crystal structure. The refinement goes through a convergence algorithm that minimizes the residuals, calculated as

where the sum is over the profile values, and *y*_*i*o_ and *y*_*i*c_ are the observed and calculated values of the profile at the *i*th position, respectively. Furthermore, *w*_*i*_ represents a weight associated with the experimental value, typically taken as the reciprocal variances of the observed intensities (Dinnebier & Billinge, 2008 ▸; Madsen *et al.*, 2019 ▸). The RR least-square procedure allows one to obtain the scale factor for each phase α, which is bound to the weight fraction by the following equation:

where *S*_α_ is the Rietveld scale factor for phase α, *W*_α_ is the weight fraction of the phase α, ρ_α_ is the density of the phase α,  is the mass absorption coefficient of the mixture, *V*_α_ is the volume of the unit cell for phase α, and *K* is defined as the ‘experiment constant’ for the instrumental setup and data-collection conditions.

Since the density can be defined as

where *ZM* is the mass of the unit-cell contents from *Z* (the number of formula units per unit cell) and *M* (the mass of the formula unit), equation (4) can be written as

*RR* is thus based on the best possible estimation of the scale factor of each phase to obtain the weight factor by equation (4). The main problem with this approach is that  and *K* are unknown, but, under the assumption that all phases in the mixture are crystalline, a simplification can be carried out according to

Equation (7) allows elimination of the instrument constants and the mass absorption coefficient (Madsen *et al.*, 2019 ▸; Dinnebier *et al.*, 2018 ▸). The presence of PO introduces inaccuracies in the scale-factor determination from the least-square procedure. March–Dollase correction or spherical harmonics functions can correct intensity for PO but great care is needed in quantitative analysis (Madsen *et al.*, 2001 ▸; Scarlett *et al.*, 2002 ▸). MA further complicates the quantification, as detailed in Section 1.1. Factors like Brindley correction can mitigate this effect but the assumption of spherical particles of identical diameter is unrealistic (Scarlett & Madsen, 2018 ▸; Pederson *et al.*, 2003 ▸).

### A3. The PONKCS method in XRPD data analysis

PONKCS (Scarlett & Madsen, 2006 ▸, 2018 ▸) was developed for the specific goal of overcoming the intrinsic limitation of RR, *i.e.* the knowledge of the crystal structures, while maintaining its formalism in the least-square minimization between experimental and theoretical profiles. It uses the knowledge of pure phase XRPD patterns for unknown phases and RR to refine known phases. The advantage over ‘pure MultiFit’ by *RootProf* (Caliandro & Belviso, 2014 ▸) is that known and unknown phases can be refined together in the same pattern, optimizing information extraction. For non-indexed phases, *ZMV* is not known, while for indexed but not solved phases, *ZM* is unknown and cell information is used to refine peak positions. In the first case, *ZMV* is estimated using a pure phase pattern to ‘calibrate’ the intensities of the phase of interest.

When α is an unknown phase, (*ZM*)_α_ is calculated using equation (8), while, when known, it is calculated as in the Rietveld approach by its crystal structure. Now (*ZM*)_α_ has no physical meaning and is only valid for the chosen experimental configuration. If the cell is known, the approach is similar, and the information is used to refine peak positions and calculate *ZM*. In both cases, the calibration procedure allows one to estimate the scale factor and can help manage MA and/or PO effects, if they are similar in pure phases and in the analysed mixture XRPD patterns. In principle, the number of PONKCS phases is arbitrary and the ideal one can be chosen to optimize the fit and phase estimation.

In this work, in order to compensate for the MA effect, the PONKCS approach was first used to generate this empirical (*ZM*)_lighter phase_ for the lighter phase [thus not exploiting the knowledge of its crystal structure, fitted by Pawley fit, using as reference the sample with 50:50 composition (*i.e.* in data set D1, S5 and S6 samples, both cases were tested) in order to get from the fit the expected 50% weight]. After this calibration step for the rest of the data set, the individual intensities of the PONKCS ‘lighter phase’ were kept fixed and only the Rietveld scale factor was left free in refinement, as done usually in standard RR. We also tested a ‘double PONKCS’ approach (one phase RR and two phases PONKCS fitted), in which both the lighter phases and one of the heavier phases, barite or bismite, were both PONKCS calibrated and Pawley fitted on the 33:33:33 mixture, to get from the fit the exact 33.3 weight percentage for each phase.
