# Supplementary material for: Multivariate versus traditional quantitative phase analysis of X-ray powder diffraction and fluorescence data of mixtures showing preferred orientation and microabsorption
Source: J Appl Crystallogr. 2022 Jul 5;55(Pt 4):837–50. doi: 10.1107/S1600576722004708 (PMC9348868; doi:10.1107/S1600576722004708)
Supplement: Supplementary file 1 [file j-55-00837-sup1.pdf]

JOURNAL OF
APPLIED
CRYSTALLOGRAPHY

Volume 55 (2022)

Supporting information for article:

Multivariate versus traditional quantitative phase analysis of X-ray powder diffraction and fluorescence data of mixtures showing preferred orientation and microabsorption

Mattia Lopresti, Beatrice Mangolini, Marco Milanesio, Rocco Caliandro and Luca Palin

Multivariate vs. traditional quantitative phase analysis of X-ray powder diffraction and fluorescence data of mixtures showing preferred orientation and microabsorption

Mattia Lopresti,[a] Beatrice Mangolini,[a] Marco Milanesio,[a]
Rocco Caliandro[b] and Luca Palin [a,c]*

[a] *Università del Piemonte Orientale, Dipartimento di Scienze e Innovazione Tecnologica, Viale T. Michel 11, 15121 Alessandria, Italy,* [b] *Institute of Crystallography, CNR, via Amendola, 122/o, 70126 Bari, Italy, and* [c] *Nova Res s.r.l., Via D. Bello 3, 28100 Novara, Italy. E-mail: luca.palin@uniupo.it*

X-ray Powder Diffraction; **Quantitative phase analysis**; **Rietveld refinement**; **Multivariate analysis**; **Principal Component Analysis**

1. Supplementary figures

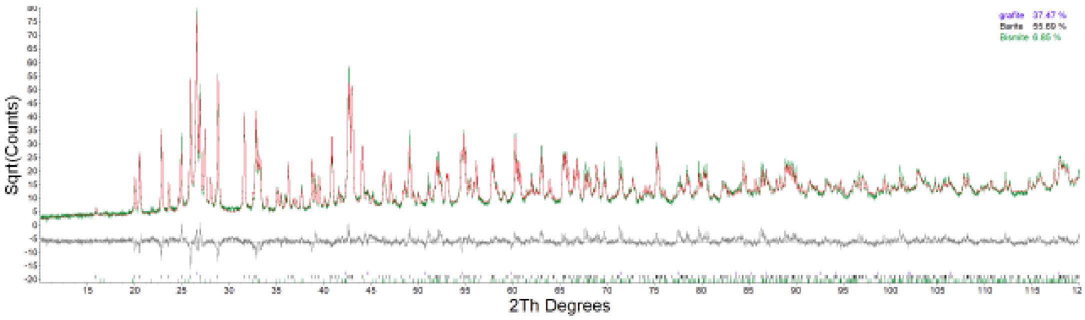

Figure S1: Whole profile fitting performed using Rietveld refinement on sample SA1 from dataset D1. The calculation of the residuals does not suggest that some parameters in the fit model have been neglected.

2. Quantification tables

Table S1: Results of the supervised analysis performed by multivariate approach.

**Dataset D1 - XRPD**			
Phase	Supervised multiple regression analysis		
	SA 1	SA 2	SA 3
Ba	0.634 (-0.032)	0.236 (0.069)	0.134 (-0.033)
Bi	0.216 (0.049)	0.577 (-0.089)	0.252 (0.085)
Gr	0.150 (-0.017)	0.188 (0.021)	0.615 (-0.051)
**Dataset D2 - XRPD**			
Phase	Supervised multiple regression analysis		
	SA 1	SA 2	SA 3
Ba	0.615 (-0.051)	0.143 (-0.024)	0.077 (-0.089)
Bi	0.137 (-0.030)	0.655 (-0.011)	0.244 (0.077)
Gr	0.248 (0.081)	0.202 (0.035)	0.678 (0.012)
**Dataset D3 - XRPD**			
Phase	Supervised multiple regression analysis		
	SA 1	SA 2	SA 3
Ba	0.608 (-0.058)	0.166 (-0.001)	0.192 (0.025)
Bi	0.156 (-0.011)	0.580 (-0.086)	0.169 (0.002)
Zn	0.236 (0.069)	0.254 (0.087)	0.638 (-0.028)
**Dataset D4 - XRPD**			
Phase	Supervised multiple regression analysis		
	SA 1	SA 2	SA 3
Ba	0.612 (-0.054)	0.209 (0.042)	0.213 (0.046)
Bi	0.183 (0.016)	0.605 (-0.061)	0.216 (0.049)
Ur	0.204 (0.037)	0.185 (0.018)	0.570 (-0.096)

Table S2: Results of the unsupervised analysis performed by multivariate approach.

			**Dataset D1 - XRPD**				
Phase	Unsupervised multiple regression analysis						
	S4 (Ba Bi)	S5 (Ba Gr)	S6 (Bi Gr)	S7 (Ba Bi Gr)	SA 1	SA 2	SA 3
Ba	0.582 (0.082)	0.367 (-0.133)	0.000 (0.000)	0.374 (0.041)	0.634 (-0.032)	0.235 (0.068)	0.133 (-0.034)
Bi	0.418 (-0.083)	0.014 (0.014)	0.581 (0.081)	0.354 (0.021)	0.215 (0.048)	0.577 (-0.089)	0.257 (0.090)
Gr	0.000 (0.000)	0.618 (0.118)	0.419 (-0.081)	0.271 (-0.062)	0.150 (-0.017)	0.188 (-0.017)	0.610 (-0.056)
			**Dataset D2 - XRPD**				
Phase	Unsupervised multiple regression analysis						
	S4 (Ba Bi)	S5 (Ba Gr)	S6 (Bi Gr)	S7 (Ba Bi Gr)	SA 1	SA 2	SA 3
Ba	0.582 (0.082)	0.436 (-0.064)	0.000 (0.000)	0.246 (-0.087)	0.615 (-0.051)	0.143 (-0.024)	0.077 (-0.090)
Bi	0.418 (-0.082)	0.000 (0.000)	0.580 (0.080)	0.390 (0.057)	0.137 (-0.030)	0.655 (-0.011)	0.244 (0.077)
Gr	0.000 (0.000)	0.564 (0.064)	0.419 (-0.081)	0.363 (0.030)	0.248 (0.081)	0.202 (0.035)	0.678 (0.012)
			**Dataset D3 - XRPD**				
Phase	Unsupervised multiple regression analysis						
	S4 (Ba Bi)	S5 (Ba Zn)	S6 (Bi Zn)	S7 (Ba Bi Zn)	SA 1	SA 2	SA 3
Ba	0.562 (0.062)	0.381 (-0.119)	0.000 (0.000)	0.385 (0.052)	0.608 (-0.058)	0.166 (-0.001)	0.192 (0.025)
Bi	0.438 (-0.062)	0.000 (0.000)	0.444 (-0.056)	0.246 (-0.054)	0.156 (-0.011)	0.580 (-0.086)	0.169 (0.002)
Gr	0.000 (0.000)	0.619 (0.119)	0.555 (0.055)	0.368 (0.035)	0.236 (0.069)	0.254 (0.087)	0.638 (-0.028)
			**Dataset D4 - XRPD**				
Phase	Unsupervised multiple regression analysis						
	S4 (Ba Bi)	S5 (Ba Ur)	S6 (Bi Ur)	S7 (Ba Bi Ur)	SA 1	SA 2	SA 3
Ba	0.545 (0.045)	0.587 (0.087)	0.000 (0.000)	0.333 (0.000)	0.607 (-0.059)	0.209 (0.042)	0.216 (0.049)
Bi	0.426 (-0.074)	0.013 (0.013)	0.376 (-0.124)	0.382 (0.049)	0.180 (0.013)	0.601 (-0.065)	0.213 (0.046)
Gr	0.028 (0.028)	0.399 (-0.101)	0.624 (0.124)	0.284 (-0.049)	0.213 (0.046)	0.189 (0.022)	0.571 (-0.096)

Table S3: Results of the blind analysis performed by multivariate approach.

					**Dataset D1 - XRPD**					
Phase	Blind analysis									
	S1 (Ba)	S2 (Bi)	S3 (Gr)	S4 (Ba Bi)	S5 (Ba Gr)	S6 (Bi Gr)	S7 (Ba Bi Gr)	SA 1	SA 2	SA 3
Ba	0.995 (-0.005)	-0.027 (-0.027)	-0.011 (-0.011)	0.366 (-0.134)	0.563 (0.063)	-0.031 (-0.031)	0.384 (0.051)	0.748 (0.081)	0.133 (-0.034)	0.255 (0.088)
Bi	0.005 (0.005)	1.000 (0.000)	0.012 (0.012)	0.575 (0.075)	0.016 (0.016)	0.384 (-0.116)	0.232 (-0.101)	0.000 (-0.167)	0.575 (-0.091)	0.111 (-0.056)
Gr	0.000 (0.000)	0.027 (0.000)	1.000 (0.000)	0.059 (0.059)	0.421 (-0.079)	0.647 (0.147)	0.384 (0.051)	0.252 (0.085)	0.292 (0.125)	0.634 (-0.032)
					**Dataset D2 - XRPD**					
Phase	Blind analysis									
	S1 (Ba)	S2 (Bi)	S3 (Gr)	S4 (Ba Bi)	S5 (Ba Gr)	S6 (Bi Gr)	S7 (Ba Bi Gr)	SA 1	SA 2	SA 3
Ba	1.000 (0.000)	-0.065 (-0.065)	-0.074 (-0.074)	0.621 (0.121)	0.382 (-0.118)	-0.042 (-0.042)	0.376 (0.043)	0.695 (0.029)	0.218 (0.051)	0.090 (-0.077)
Bi	0.000 (0.000)	1.000 (0.000)	0.074 (0.074)	0.368 (-0.132)	0.046 (0.046)	0.546 (0.046)	0.343 (0.010)	0.137 (-0.030)	0.645 (-0.021)	0.233 (-0.100)
Gr	0.000 (0.000)	0.065 (0.000)	1.000 (0.000)	0.011 (0.011)	0.572 (0.072)	0.496 (0.004)	0.281 (-0.115)	0.168 (0.001)	0.137 (-0.030)	0.677 (0.011)
					**Dataset D3 - XRPD**					
Phase	Blind analysis									
	S1 (Ba)	S2 (Bi)	S3 (Zn)	S4 (Ba Bi)	S5 (Ba Zn)	S6 (Bi Zn)	S7 (Ba Bi Zn)	SA 1	SA 2	SA 3
Ba	0.847 (-0.153)	-0.087 (-0.087)	0.000 (0.000)	0.468 (0.032)	0.432 (0.068)	-0.050 (-0.050)	0.246 (0.087)	0.500 (-0.166)	0.167 (0.000)	0.111 (-0.056)
Bi	0.000 (0.000)	0.087 (0.087)	1.000 (0.000)	0.445 (-0.055)	0.029 (0.029)	0.482 (-0.018)	0.290 (-0.043)	0.202 (0.035)	0.610 (-0.056)	0.229 (0.062)
Zn	0.153 (0.153)	1.000 (0.000)	0.000 (0.000)	0.087 (0.000)	0.540 (0.040)	0.569 (0.069)	0.465 (0.132)	0.300 (0.133)	0.223 (0.056)	0.660 (-0.006)
					**Dataset D4 - XRPD**					
Phase	Blind analysis									
	S1 (Ba)	S2 (Bi)	S3 (Ur)	S4 (Ba Bi)	S5 (Ba Ur)	S6 (Bi Ur)	S7 (Ba Bi Ur)	SA 1	SA 2	SA 3
Ba	0.954 (-0.046)	0.000 (0.000)	0.000 (0.000)	0.453 (-0.047)	0.236 (-0.214)	0.003 (0.003)	0.181 (-0.152)	0.408 (-0.254)	0.091 (0.076)	0.028 (-0.139)
Bi	0.006 (0.006)	0.001 (0.001)	1.000 (0.000)	0.464 (0.036)	0.000 (0.000)	0.429 (0.071)	0.365 (0.032)	0.232 (0.065)	0.667 (0.001)	0.190 (0.023)
Ur	0.040 (0.040)	1.000 (0.000)	0.000 (0.000)	0.084 (0.084)	0.764 (0.264)	0.569 (0.069)	0.454 (0.121)	0.360 (0.193)	0.242 (0.075)	0.783 (0.117)

Table S4: Results of the quantitative phase analysis performed by Rietveld refinement.

**Dataset D1 - XRPD**							
Phase	Rietveld refinement						
	S4 (Ba Bi)	S5 (Ba Gr)	S6 (Bi Gr)	S7 (Ba Bi Gr)	SA 1	SA 2	SA 3
Ba	0.767 (0.267)	0.139 (-0.361)	0.000 (0.000)	0.293 (-0.040)	0.556 (-0.110)	0.207 (0.040)	0.044 (-0.123)
Bi	0.233 (-0.267)	0.000 (0.000)	0.189 (-0.311)	0.140 (-0.193)	0.069 (-0.098)	0.336 (-0.330)	0.037 (-0.130)
Gr	0.000 (0.000)	0.861 (0.361)	0.811 (0.311)	0.567 (0.234)	0.375 (0.208)	0.457 (0.290)	0.919 (0.253)

**Dataset D2 - XRPD**							
Phase	Rietveld refinement						
	S4 (Ba Bi)	S6 (Bi Gr)	S7 (Ba Bi Gr)	SA 1	SA 2	SA 3	
Ba	0.767 (0.267)	0.153 (-0.347)	0.000 (0.000)	0.175 (-0.158)	0.491 (-0.175)	0.108 (0.013)	0.029 (-0.138)
Bi	0.233 (-0.267)	0.000 (0.000)	0.188 (-0.312)	0.150 (-0.183)	0.079 (-0.088)	0.383 (-0.283)	0.044 (-0.123)
Gr	0.000 (0.000)	0.847 (0.347)	0.812 (0.312)	0.675 (0.342)	0.430 (0.263)	0.509 (0.342)	0.927 (0.261)

**Dataset D3 - XRPD**							
Phase	Rietveld refinement						
	S4 (Ba Bi)	S6 (Bi Zn)	S7 (Ba Bi Zn)	SA 1	SA 2	SA 3	
Ba	0.767 (0.267)	0.377 (-0.123)	0.000 (0.000)	0.430 (0.097)	0.654 (-0.012)	0.238 (0.071)	0.175 (0.008)
Bi	0.233 (-0.267)	0.000 (0.000)	0.200 (-0.300)	0.122 (-0.211)	0.064 (-0.103)	0.342 (-0.324)	0.061 (-0.106)
Zn	0.000 (0.000)	0.623 (0.123)	0.800 (0.000)	0.448 (0.115)	0.282 (0.115)	0.420 (0.253)	0.764 (0.098)

**Dataset D4 - XRPD**							
Phase	Rietveld refinement						
	S4 (Ba Bi)	S6 (Bi Ur)	S7 (Ba Bi Ur)	SA 1	SA 2	SA 3	
Ba	0.767 (0.267)	0.471 (-0.129)	0.000 (0.000)	0.525 (0.192)	0.749 (0.083)	0.263 (0.096)	0.190 (0.023)
Bi	0.233 (-0.267)	0.000 (0.000)	0.149 (-0.351)	0.168 (-0.165)	0.075 (-0.092)	0.322 (-0.344)	0.058 (-0.109)
Ur	0.000 (0.000)	0.529 (0.129)	0.851 (0.351)	0.308 (-0.025)	0.176 (0.009)	0.415 (0.248)	0.752 (0.086)

Table S5: Results of the quantitative phase analysis performed by single PONKCS approach.

Dataset D1 - XRPD						
Phase	Single PONKCS on barite					
	S4 (Ba Bi)	S6 (Bi Gr)	S7 (Ba Bi Gr)	SA 1	SA 2	SA 3
Ba	0.767 (0.267)	0.000 (0.000)	0.542 (0.209)	0.760 (0.094)	0.300 (0.133)	0.171 (0.004)
Bi	0.233 (-0.267)	0.522 (0.022)	0.243 (-0.090)	0.091 (-0.076)	0.448 (-0.218)	0.132 (-0.035)
Gr	0.000 (0.000)	0.478 (-0.022)	0.215 (-0.118)	0.149 (-0.018)	0.252 (0085)	0.698 (0.032)
Phase	Single PONKCS on bismite					
	S4 (Ba Bi)	S5 (Ba Gr)	S7 (Ba Bi Gr)	SA 1	SA 2	SA 3
Ba	0.767 (0.267)	0.517 (0.017)	0.489 (0.156)	0.722 (0.056)	0.266 (0.099)	0.133 (-0.034)
Bi	0.233 (-0.267)	0.000 (0.000)	0.252 (-0.081)	0.099 (-0.068)	0.439 (-0.222)	0.128 (-0.039)
Gr	0.000 (0.000)	0.483 (-0.017)	0.259 (-0.074)	0.179 (0.012)	0.294 (0.127)	0.739 (0.073)
**Dataset D2 - XRPD**						
Phase	Single PONKCS on barite					
	S4 (Ba Bi)	S6 (Bi Gr)	S7 (Ba Bi Gr)	SA 1	SA 2	SA 3
Ba	0.767 (0.267)	0.000 (0.000)	0.416 (0.083)	0.606 (-0.060)	0.191 (0.024)	0.098 (-0.069)
Bi	0.233 (-0.267)	0.454 (-0.046)	0.288 (-0.045)	0.088 (-0.079)	0.546 (-0.120)	0.119 (-0.048)
Gr	0.000 (0.000)	0.546 (0.046)	0.296 (-0.037)	0.306 (0.139)	0.263 (0.096)	0.793 (0.127)
Phase	Single PONKCS on bismite					
	S4 (Ba Bi)	S5 (Ba Gr)	S7 (Ba Bi Gr)	SA 1	SA 2	SA 3
Ba	0.767 (0.267)	0.557 (0.057)	0.442 (0.109)	0.608 (-0.058)	0.198 (0.031)	0.108 (-0.059)
Bi	0.233 (-0.267)	0.000 (0.000)	0.306 (-0.027)	0.087 (-0.080)	0.573 (-0.093)	0.131 (-0.036)
Gr	0.000 (0.000)	0.443 (-0.057)	0.253 (-0.080)	0.305 (0.138)	0.229 (0.062)	0.761 (0.095)
**Dataset D3 - XRPD**						
Phase	Single PONKCS on barite					
	S4 (Ba Bi)	S6 (Bi Zn)	S7 (Ba Bi Zn)	SA 1	SA 2	SA 3
Ba	0.767 (0.267)	0.000 (0.000)	0.558 (-0.108)	0.706 (0.040)	0.281 (0.114)	0.240 (0.037)
Bi	0.233 (-0.267)	0.482 (-0.018)	0.156 (-0.177)	0.073 (-0.094)	0.417 (-0.249)	0.092 (-0.075)
Zn	0.000 (0.000)	0.518 (0.018)	0.286 (-0.047)	0.221 (0.054)	0.302 (0.135)	0.668 (0.002)
Phase	Single PONKCS on bismite					
	S4 (Ba Bi)	S5 (Ba Zn)	S7 (Ba Bi Zn)	SA 1	SA 2	SA 3
Ba	0.767 (0.267)	0.654 (0.154)	0.626 (0.293)	0.800 (0.134)	0.343 (0.176)	0.382 (0.215)
Bi	0.233 (-0.267)	0.000 (0.000)	0.159 (-0.174)	0.070 (-0.097)	0.444 (-0.222)	0.095 (-0.072)
Zn	0.000 (0.000)	0.346 (-0.154)	0.215 (-0.118)	0.130 (-0.037)	0.213 (0.046)	0.523 (-0.143)
**Dataset D4 - XRPD**						
Phase	Single PONKCS on barite					
	S4 (Ba Bi)	S6 (Bi Ur)	S7 (Ba Bi Ur)	SA 1	SA 2	SA 3
Ba	0.767 (0.267)	0.000 (0.000)	0.617 (0.284)	0.831 (0.165)	0.312 (0.145)	0.262 (0.095)
Bi	0.233 (-0.267)	0.169 (-0.331)	0.185 (-0.148)	0.073 (-0.094)	0.371 (-0.295)	0.080 (-0.087)
Ur	0.000 (0.000)	0.831 (0.331)	0.199 (-0.134)	0.096 (-0.071)	0.317 (0.150)	0.658 (-0.008)
Phase	Single PONKCS on bismite					
	S4 (Ba Bi)	S5 (Ba Ur)	S7 (Ba Bi Ur)	SA 1	SA 2	SA 3
Ba	0.767 (0.267)	0.898 (0.398)	0.738 (0.405)	0.904 (0.238)	0.427 (0.260)	0.598 (0.431)
Bi	0.233 (-0.267)	0.000 (0.000)	0.223 (-0.110)	0.081 (-0.086)	0.513 (-0.153)	0.174 (0.007)
Ur	0.000 (0.000)	0.102 (-0.398)	0.040 (-0.293)	0.015 (-0.152)	0.060 (-0.107)	0.228 (-0.438)

Table S6: Results of the quantification analyses performed on XRPD data sets. On the left, quantification was made by double PONKCS on barite. On the right, double PONKCS on bismite was used.

**Dataset D1 - XRPD**						
Phase	Double PONKCS on barite			Double PONKCS on bismite		
	SA 1	SA 2	SA 3	SA 1	SA 2	SA 3
Ba	0.536 (-0.130)	0.140 (-0.027)	0.105 (-0.017)	0.493 (-0.173)	0.200 (0.033)	0.206 (0.039)
Bi	0.147 (-0.020)	0.507 (-0.159)	0.181 (0.014)	0.146 (-0.021)	0.477 (-0.189)	0.130 (-0.037)
Gr	0.317 (0.150)	0.353 (0.186)	0.715 (0.049)	0.361 (0.194)	0.323 (0.156)	0.665 (-0.001)
**Dataset D2 - XRPD**						
Phase	Double PONKCS on barite			Double PONKCS on bismite		
	SA 1	SA 2	SA 3	SA 1	SA 2	SA 3
Ba	0.512 (-0.154)	0.139 (-0.028)	0.118 (-0.049)	0.434 (-0.232)	0.151 (-0.016)	0.135 (-0.032)
Bi	0.098 (-0.069)	0.563 (-0.103)	0.186 (0.019)	0.083 (-0.084)	0.591 (-0.075)	0.155 (-0.012)
Gr	0.391 (0.224)	0.298 (0.0131)	0.696 (0.030)	0.484 (0.317)	0.258 (0.091)	0.710 (0.044)
**Dataset D3 - XRPD**						
Phase	Double PONKCS on barite			Double PONKCS on bismite		
	SA 1	SA 2	SA 3	SA 1	SA 2	SA 3
Ba	0.504 (-0.162)	0.120 (-0.047)	0.131 (-0.036)	0.533 (-0.133)	0.118 (-0.049)	0.148 (-0.019)
Bi	0.191 (0.024)	0.611 (-0.055)	0.174 (0.007)	0.185 (0.018)	0.662 (-0.004)	0.193 (0.026)
Zn	0.306 (0.139)	0.269 (0.102)	0.696 (0.030)	0.283 (0.116)	0.220 (0.053)	0.659 (-0.007)
**Dataset D4 - XRPD**						
Phase	Double PONKCS on barite			Double PONKCS on bismite		
	SA 1	SA 2	SA 3	SA 1	SA 2	SA 3
Ba	0.593 (-0.073)	0.116 (-0.051)	0.126 (-0.041)	0.582 (-0.084)	0.135 (-0.032)	0.135 (-0.032)
Bi	0.197 (-0.030)	0.432 (-0.234)	0.108 (0.059)	0.170 (0.003)	0.518 (-0.148)	0.125 (-0.042)
Ur	0.209 (0.042)	0.450 (0.283)	0.765 (0.099)	0.248 (0.081)	0.347 (0.180)	0.740 (0.074)

Table S7: Results of the quantification analyses performed on XRF data set D3. On the left, quantification was made by fundamental parameters approach. On the right, supervised multiple regression analysis was used.

**Dataset D3 - XRF**						
Phase	FP			SMRA		
	SA 1	SA 2	SA 3	SA 1	SA 2	SA 3
Ba	0.523 (-0.143)	0.144 (-0.023)	0.332 (0.165)	0.623 (-0.043)	0.214 (0.047)	0.163 (-0.004)
Bi	0.152 (-0.015)	0.668 (0.002)	0.179 (0.012)	0.158 (-0.009)	0.553 (-0.113)	0.093 (-0.074)
Zn	0.129 (-0.038)	0.165 (-0.002)	0.706 (-0.04)	0.280 (0.113)	0.216 (0.049)	0.504 (-0.162)
